# Calcineurin signaling promotes takotsubo syndrome

**DOI:** 10.1038/s44161-023-00296-w

**Published:** 2023-07-13

**Authors:** Bastian Bruns, Marilena Antoniou, Irena Baier, Maximilian Joos, Meryem Sevinchan, Marie-Christine Moog, Christoph Dieterich, Hans-Christoph Friederich, Hilal Khan, Heather Wilson, Wolfgang Herzog, Dana K. Dawson, Norbert Frey, Jobst-Hendrik Schultz, Johannes Backs

**Affiliations:** 1 Institute of Experimental Cardiology, Heidelberg University Hospital, Heidelberg, Germany; 2 Department of Cardiology, Angiology and Pneumology, Heidelberg University Hospital, Heidelberg, Germany; 3 Department of General Internal Medicine and Psychosomatics, Heidelberg University Hospital, Heidelberg, Germany; 4 DZHK (German Centre for Cardiovascular Research), Partner Site, Heidelberg/Mannheim, Heidelberg, Germany; 5 Klaus Tschira Institute for Integrative Computational Cardiology, University Hospital Heidelberg, Heidelberg, Germany; 6 Cardiology Research Group, Aberdeen Cardiovascular and Diabetes Centre, School of Medicine and Dentistry, University of Aberdeen, Aberdeen, UK

**Keywords:** Cardiovascular diseases, Molecular biology

## Abstract

Takotsubo syndrome (TTS) is an acute heart failure syndrome that mimics the symptoms of acute myocardial infarction and is often preceded by emotional and/or physical stress. There is currently no treatment for TTS. Here we show that injection of 2.5 mg kg^−1^ of epinephrine (EPI) into mice recapitulates numerous features of human TTS, including increased myocardial damage and mortality in males. Gene set enrichment analysis of myocardial RNA sequencing after EPI injection revealed significant enrichment of calcineurin-dependent pro-inflammatory gene networks, which was more pronounced in male than in female mice, in agreement with observed sex discrepancies in the mouse phenotype. An increase in calcineurin activity was detected in the circulating cells of patients with TTS, suggesting a systemic nature of the syndrome. Preventive and therapeutic treatment of mice injected with EPI using calcineurin inhibitors cyclosporine and tacrolimus improved heart function and reduced myocardial injury. Our findings suggest that calcineurin inhibition could be a potential therapy for TTS.

## Abstract (web summary)

The ‘Broken-heart’ or takotsubo syndrome (TTS) causes acute heart failure triggered by emotional or physical stress. Bruns et al. establish a clinically relevant mouse model of TTS and show the therapeutic potential of calcineurin inhibition in the treatment of TTS.

## Main

TTS is an acute heart failure (AHF) syndrome that mimics the symptoms of acute myocardial infarction and is often preceded by an episode of severe emotional and/or physical stress^1^. The name ‘takotsubo’ stems from the first official description in which the syndrome was named after the ballooned apical shape of the affected left ventricle, resembling a Japanese octopus trap (Tako-Tsubo)^2^. Although several acute complications of TTS, such as cardiogenic shock or arrhythmias, can be life-threatening, left ventricular ejection fraction (LVEF) mostly recovers in survivors. Nevertheless, affected patients have a worse long-term prognosis^3^. Approximately 90% of cases are seen in female patients, most of whom are postmenopausal^4^, but morbidity and mortality are substantially lower in female patients than male patients^5^. Since catecholamine storm^6^, triggered by sympathetic nervous system activation^7,8^, has been suggested to have a pivotal role in the pathophysiology of TTS, the treatment of cardiogenic shock poses a particularly difficult clinical situation. The catecholamine most frequently associated with accidental induction of TTS in humans is the endogenous α- and β-adrenoceptor agonist EPI^9,10^. Experimentally, high doses of catecholamines induce transient AHF in rats and other species^11^. Mechanistically, the potential molecular causes of TTS include a switch in G protein coupling of β_2_-adrenoceptor (from coupling to stimulatory Gα_s_-protein to coupling to inhibitory Gα_i_-protein)^12^, myocardial lipid accumulation^13^, energy deficit^14^, as well as systemic and myocardial inflammation^15^. As beta blocker therapy has not proven beneficial^4^, the lack of a specific treatment strategy highlights the importance of mechanistic studies as a prerequisite for tailored therapies. Catecholamine stimulation of cardiomyocytes activates the protein phosphatase calcineurin^16,17^. Calcineurin activation has been shown to contribute primarily to cardiac hypertrophy^18^ but also to inflammation and heart failure by activating the nuclear factor of activated T cells (NFAT)^19^. Pharmacological calcineurin inhibition with cyclosporine A (CsA) is used to suppress organ rejection (for example, after heart transplantation) but has not been investigated as an anti-inflammatory approach to combat heart disease.

## Results

### Epinephrine-induced reversible AHF in mice

**Fig. 1:**
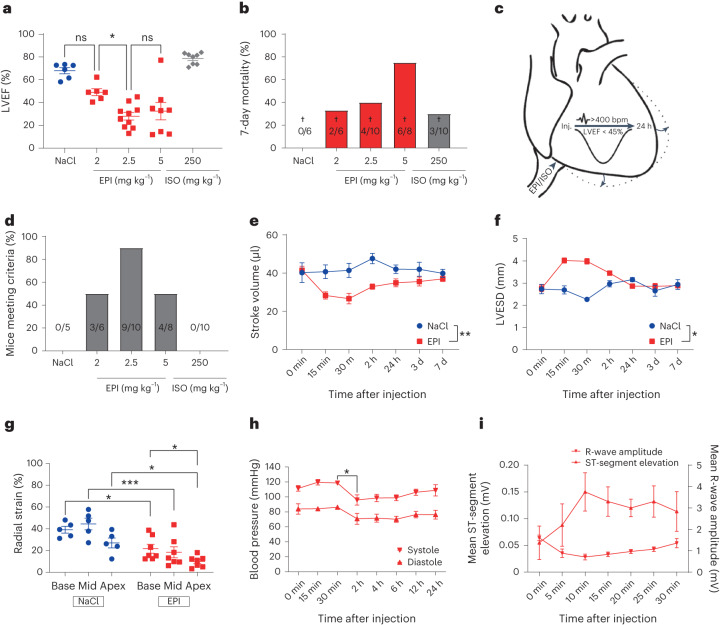
EPI-induced reversible AHF in mice. **a**, LVEF of mice undergoing isoflurane narcosis 30 min after the administration of NaCl (*n* = 6), ascending doses of EPI (EPI 2 mg kg^−1^, *n* = 6; EPI 2.5 mg kg^−1^, *n* = 10; EPI 5 mg kg^−1^, *n* = 8) or isoprenaline (ISO) 250 mg kg^−1^ (*n* = 8) (*P = 0.01). ns, not significant. **b**, Seven-day mortality with number of deceased given within bars (NaCl, *n* = 6; EPI 2 mg kg^−1^, *n* = 6; EPI 2.5 mg kg^−1^, *n* = 10; EPI 5 mg kg^−1^, *n* = 8; ISO 250 mg kg^−1^, *n* = 8). **c**, Model criteria. **d**, Percentage of surviving mice meeting criteria with number of mice given within bars (NaCl, *n* = 6; EPI 2 mg kg^−1^, *n* = 6; EPI 2.5 mg kg^−1^, *n* = 10; EPI 5 mg kg^−1^, *n* = 8; ISO 250 mg kg^−1^, *n* = 8). **e**,**f**, Echocardiography-derived left ventricular stroke volume (**P = 0.004) (**e**) and left ventricular end-systolic diameter (LVESD) timecourse (*P = 0.04) (NaCl, *n* = 5; EPI, *n* = 10) (**f**). **g**, Basal (base), midventricular (mid) and apical (apex) radial strain (NaCl, *n* = 5; EPI, *n* = 7; EPI base versus apex *P = 0.03, ***P = 0.0004). **h**, Kinetics of systolic and diastolic blood pressure (*n* = 3 in each group, *P = 0.03). **i**, ST-segment elevation and R-wave amplitude changes after EPI (*n* = 5 in each group). All mice were male and aged 8–10 weeks. Data expressed as mean ± s.e.m., from multiple comparisons adjusted ANOVA, two-sided *t*-test (**h**), two-sided paired *t*-test (15 min–7 d) (**e**) and one-tailed paired *t*-test (15 min–2 h) (**f**). Source data

**Extended Data Fig. 1:**
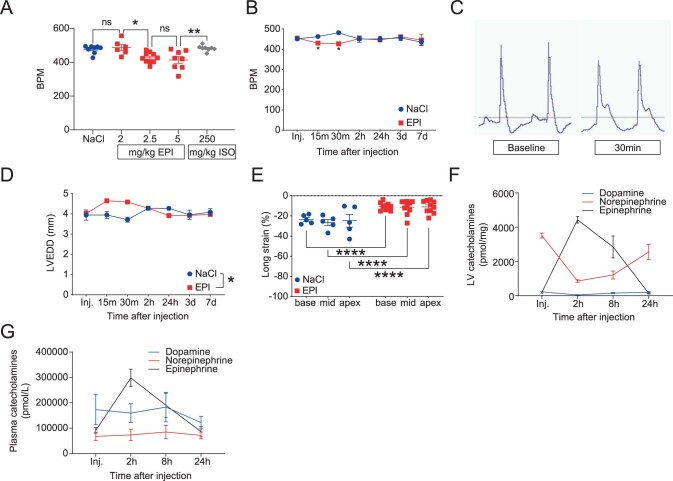
Epinephrine-induced reversible acute heart failure in mice. **(A)** Heart rate (beats per minute (BPM)) 30 min. upon NaCl 0.9% (NaCl), ascending doses of epinephrine (EPI) (2, 2.5, and 5 mg/kg), or isoprenaline (ISO) (250 mg/kg) from mice undergoing isoflurane narcosis (n = NaCl, EPI 5 mg/kg, ISO 250 mg/kg 8/group, EPI 2 mg/kg 6, EPI 2.5 mg/kg 10; *p = 0.0205, **p = 0.0045) and **(B)** time course of heart rate after 2.5 mg/kg EPI or NaCl (n = NaCl 5, EPI 10; *p = 0.045). **(C)** Representative ECG images before (Baseline) and 30 minutes (30 min.) after EPI. **(D)** Left ventricular enddiastolic diameter (LVEDD) kinetics after 2.5 mg/kg EPI (n = NaCl 5, EPI 10; at 15–30 min *p = 0.0224). **(E)** Impaired basal (base), midventricular (mid), and apical (apex) longitudinal strain upon EPI vs. NaCl in male mice at 30 min. (n = NaCl 5, EPI 10; ****p < 0.0001). **(F)** Left ventricular tissue (LV) catecholamine (dopamine, norepinephrine, epinephrine) kinetics in male mice after EPI. **(G)** Plasma catecholamine kinetics upon EPI. All mice were male (8–10w). Data as mean ± s.e.m. Multiple comparisons adjusted ANOVA (A, E) or two-sided T-test (B, D). Source data

Given that approximately threefold higher plasma levels of EPI were observed in patients with TTS than in patients with acute myocardial infarction^6^ and due to the exacerbated phenotype in men^5^, male mice were injected with increasing doses of EPI (2.0, 2.5 and 5.0 mg kg^−1^ body weight) under narcosis. Mice injected with EPI displayed a significant reduction of LVEF after 30 min compared to those injected with 0.9% NaCl, as well as substantially increased mortality (Fig. 1a,b). Notably, isoprenaline, at a comparable dose of 250 mg kg^−1^, caused a hypercontractile phenotype with no relevant mortality. We defined model criteria as a heart rate > 400 bpm and a LVEF < 45% to ensure meaningful AHF and exclude bradycardia-triggered impairment of cardiac function^20^ (Fig. 1c). A dose of 2.5 mg kg^−1^ EPI was identified as the optimal dose to facilitate reversible AHF, with 90% of C57BL/6N mice meeting model criteria (Fig. 1d and Extended Data Fig. 1a–e). Echocardiographic characterization confirmed reduced stroke volume (Fig. 1e) and ventricular ballooning (Fig. 1f and Extended Data Fig. 1d) with increased apical impairment of contractility by means of radial strain (Fig. 1g). Moreover, we observed a marked decrease in invasively measured systolic and diastolic blood pressure at 2 h with slow recovery thereafter, suggestive of incipient cardiogenic shock (Fig. 1h). ECG monitoring revealed blunted R-wave amplitude, suggestive of myocardial edema, and ST-segment elevation in the EPI-induced AHF (eTTS) model (Fig. 1i and Extended Data Fig. 1c), which are also typical findings in patients with TTS^21^. Moreover, mice displayed significantly increased plasma high-sensitive troponin T (hs-TnT) levels but lower than in myocardial infarction^22^^,23^, similarly to patients with TTS^4^.

**Fig. 2:**
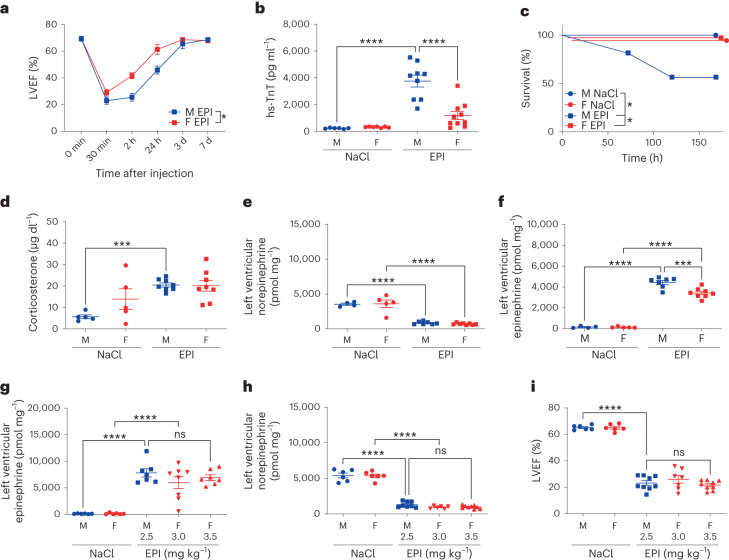
Sex-specific outcome and myocardial inflammation in eTTS. **a**, Kinetics of LVEF in 10-week-old male (M) versus female (F) mice after EPI (M: *n* = 10, F: *n* = 10; 2 h–24 h *P = 0.01). **b**, Plasma hs-TnT at 24 h in male versus female mice after NaCl or EPI (M NaCl, *n* = 6; F NaCl, *n* = 7; M EPI, *n* = 9; F EPI, *n* = 10; ****P < 0.0001). **c**, Kaplan-Maier analysis of survival of male versus female mice after NaCl or EPI (M NaCl, *n* = 5; F NaCl, *n* = 5; M EPI, *n* = 16; F EPI, *n* = 9; *P = 0.01). **d**, Plasma corticosterone levels of male versus female mice after NaCl or EPI (M NaCl, *n* = 5; F NaCl, *n* = 5; M EPI, *n* = 8; F EPI, *n* = 8; ***P = 0.0005). **e**,**f**, Left ventricular norepinephrine (**e**) and EPI (**f**) 2 h after NaCl or EPI (M NaCl, *n* = 4; F NaCl, *n* = 5; M EPI, *n* = 7; F EPI, *n* = 8; ****P < 0.0001 and ***P = 0.0002). **g**, Left ventricular EPI after NaCl or ascending doses of EPI (M NaCl, *n* = 6; F NaCl, *n* = 6; M EPI 2.5 mg kg^−1^, *n* = 7; F EPI 3.0 mg kg^−1^, *n* = 8; F EPI 3.5 mg kg^−1^, *n* = 7; ****P < 0.0001). **h**, Left ventricular norepinephrine after NaCl or ascending doses of EPI (M NaCl, *n* = 6; F NaCl, *n* = 6; M EPI 2.5 mg kg^−1^, *n* = 8; F EPI 3.0 mg kg^−1^, *n* = 6; F EPI 3.5 mg kg^−1^, *n* = 9; ****P < 0.0001). **i**, LVEF of 12-week-old male mice treated with NaCl (M NaCl) or 2.5 mg kg^−1^ EPI (M EPI) compared to female mice treated with NaCl (F NaCl) or ascending doses of EPI (F EPI) (M NaCl, *n* = 6; F NaCl, *n* = 6; M EPI 2.5 mg kg^−1^, *n* = 9; F EPI 3.0 mg kg^−1^, *n* = 7; F EPI 3.5 mg kg^−1^, *n* = 9; ****P < 0.0001). Data expressed as mean ± s.e.m., from multiple comparisons adjusted ANOVA or two-sided paired *t*-test (**a**). Source data

### Sex-specific outcome and myocardial inflammation in eTTS

**Extended Data Fig. 2:**
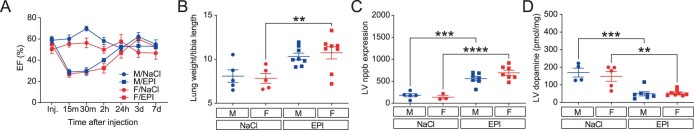
Gender- and age discrepancies in epinephrine-induced heart failure. **(A)** Left ventricular ejection fraction (EF%) kinetics over 7d from 10w old male (M) and female mice (F), after NaCl 0.9% (NaCl) or epinephrine (EPI) injection (Inj.) under isoflurane narcosis (n = M/NaCl, F/NaCl 6/group, M/EPI 10, F/EPI 9). **(B)** Lung weight per tibia length (n = M/NaCl, F/NaCl 5/group, M/EPI, F/EPI 8/group; **p = 0.003) and **(C)** LV nppb/gapdh mRNA expression 2 h after NaCl or EPI treatment in M vs. F mice (n = M/NaCl 5, M/EPI, F/EPI 7/group, F/NaCl 3; ***p = 0.0001). **(D)** Sex comparison of LV dopamine at 2 h (n = M/NaCl 4, M/EPI 7, F/NaCl 5, F/EPI 8; **p = 0.0026, ***p = 0.0005). Data as mean ± SEM. Multiple comparisons adjusted ANOVA (B–D). Source data

Given that male patients with TTS have worse outcomes than female patients, we next compared male and female C57BL/6N mice injected with NaCl or EPI. Male patients with TTS suffer from increased myocardial damage, complications and mortality^24^, and this was recapitulated in male mice injected with EPI, which had reduced LVEF (observed with awake echocardiography (Fig. 2a) but masked under narcosis (Extended Data Fig. 2a–d)), significantly increased hs-TnT (Fig. 2b), as well as increased mortality (Fig. 2c) compared to female mice injected with EPI. Moreover, increased plasma corticosterone, the murine analog of cortisol in humans, revealed comparable secondary activation of the central hypothalamo-pituitary-adrenal (HPA) axis (also known as the stress axis) (Fig. 2d) and blunted left ventricular tissue norepinephrine, suggesting cardiac sympathetic nervous system activation (Fig. 2e and Extended Data Fig. 1f) in male and female mice. However, plasma (Extended Data Fig. 1g) and left ventricular tissue EPI were increased 2 h after injection (Fig. 2f and Extended Data Fig. 1f). This increase was significantly higher in male mice, pointing possibly to sex-dependent degradation mechanisms. In female mice, higher doses of EPI were required than in male mice for similar levels of left ventricular tissue EPI (Fig. 2g), norepinephrine (Fig. 2h) and LVEF (Fig. 2i).

**Fig. 3:**
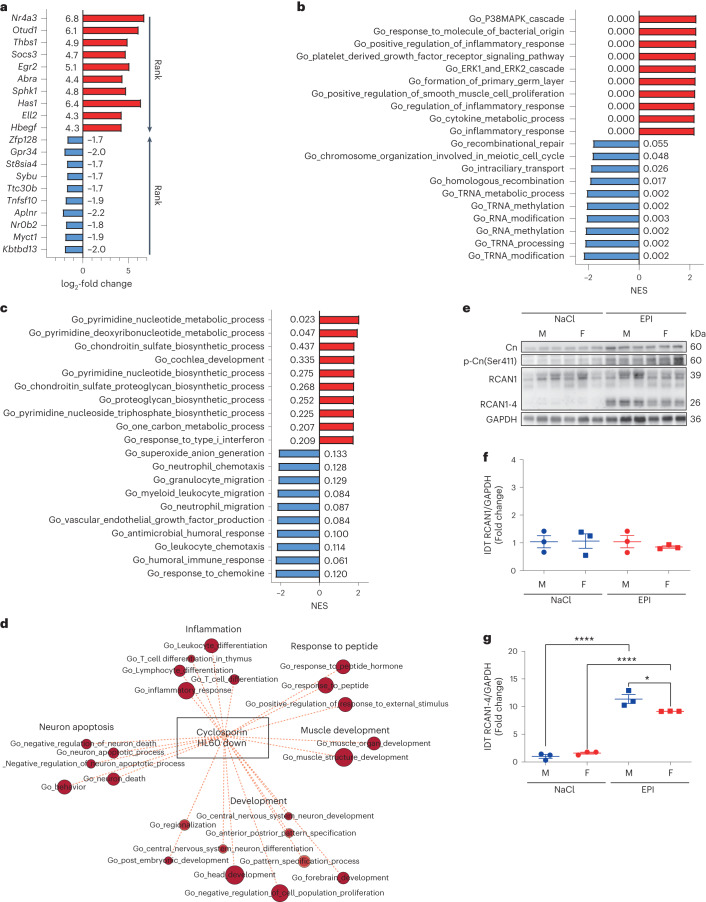
Sex-specific calcineurin-driven inflammation. **a**–**c**, GSEA from RNA sequencing performed in left ventricular tissue of 10-week-old mice treated with NaCl and EPI. **a**, log_2_-fold change of top-ranked upregulated (red) and downregulated (blue) genes of male mice treated with NaCl (*n* = 5) vs. EPI (n = 7) 2 h after the injection. **b**, Normalized enrichment score (NES) and false discovery rate (FDR (numbers next to bars)) of top-ranked upregulated (red) and downregulated (blue) enriched gene set ontology (GO) biological pathways of male mice treated with NaCl (*n* = 5) versus EPI (*n* = 7). **c**, NES and FDR of top-ranked upregulated (red) and downregulated (blue) enriched GO biological pathways of female (*n* = 7) versus male (*n* = 7) mice 2 h after EPI. **d**, Enrichment map illustrating discrepantly regulated overlapping myocardial pathways, upregulated in male mice treated with EPI and downregulated by the calcineurin inhibitor CsA, based on the Tanlab drug signature database gene set Cyclosporin_HL60_DOWN (*P* = 0.0174). **e**, Immunoblotting of calcineurin (Cn), serine 411-phospho-Cn (p-Cn(Ser411)), RCAN1 and RCAN1-4, as well as GAPDH from left ventricular tissue 2 h after NaCl or EPI in male versus female mice. **f**,**g**, Immunoblotting integrated density (IDT) protein quantification of RCAN1/GAPDH (**f**) and RCAN1-4/GAPDH (**g**) (*n* = 3 in each group, *P = 0.021, ****P *<* 0.0001). Data expressed as mean ± s.e.m., from multiple comparisons adjusted ANOVA or two-sided Mann–Whitney *U*-test (**d**). Source data

**Extended Data Fig. 3:**
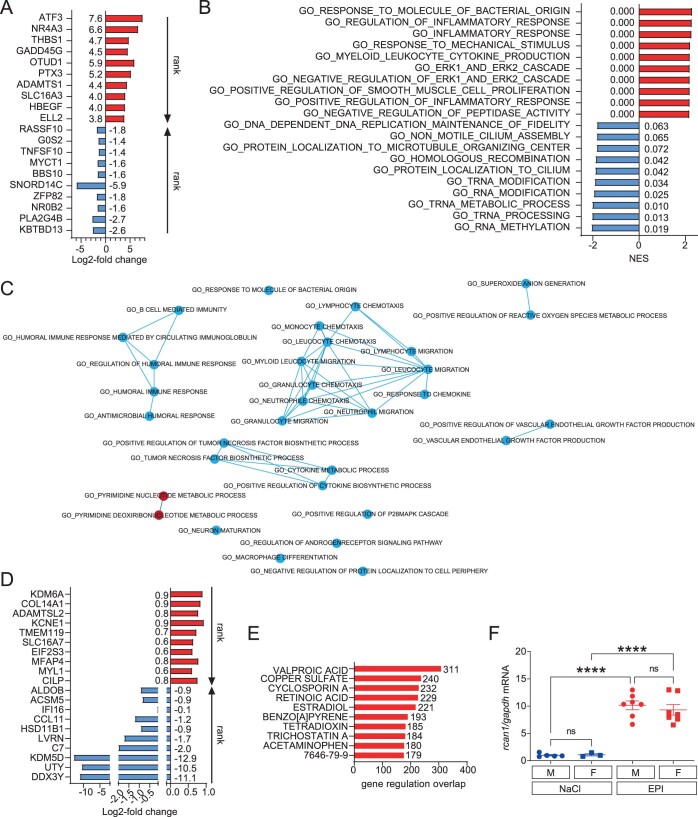
Epinephrine-induced heart failure promotes pro-inflammatory myocardial gene expression networks. **(A, B)** Gene set enrichment analysis (GSEA) from RNA-sequencing (n = M/NaCl 5, F/NaCl 3, M/EPI, F/EPI 7/group) was conducted in NaCl- vs. epinephrine (EPI)-treated 10w old female as well as in **(C, D)** EPI-treated female vs. male mice from left ventricular tissue (LV). **(A)** Log2-fold change of top ranked up- (red) and downregulated (blue) genes of NaCl vs. EPI-treated females, as well as of **(D)** EPI-treated females vs. males 2 h after insult. **(B)** Normalized enrichment score (NES) and – next to bars – false discovery rate (FDR) of top up- (red) and downregulated (blue) enriched gene set ontology (GO) biological pathways of NaCl vs. EPI-treated female mice 2 h after insult. **(C)** Intersection network of GSEA enrichment map depicting significant positive (red), and negative (blue) enriched GO biological pathways of female vs. male mice after EPI. Each node depicts one GO biological pathway gene set with connecting line thickness accounting for the number of common genes per pathway. **(E)** Top ten drug targets with significant myocardial gene expression overlap of NaCl vs. epinephrine (EPI)-treated male mice from the Tanlab drug signature database (*p = 0.0003). **(F)** Quantification of regulator of calcineurin 1 (rcan1) mRNA from 12w old male and female mice after NaCl vs. EPI (n = M/NaCl, F/NaCl, F/EPI 4/group, M/EPI 5; ****p < 0.0001). Data as mean ± s.e.m. Two-sided Mann-Whitney test (E) and multiple comparisons adjusted ANOVA (F). Source data

As an unbiased assessment of sex-dependent and independent pathways after EPI, we conducted gene set enrichment analysis (GSEA) from RNA sequencing data of left ventricular tissue from male and female mice with and without eTTS (Fig. 3a–d and Extended Data Fig. 3a–d). The discrepancies between male and female mice in EPI-induced heart failure were less upregulation of pro-inflammatory (cytokine biosynthesis, lymphocyte and neutrophil chemotaxis), VEGF production and p38 MAPK pathway gene sets in female mice, possibly explaining the reduced myocardial damage observed in this model (Fig. 3b,c). However, because myocardial damage and inflammation are reciprocal processes, increased myocardial damage in male mice may also contribute to increased inflammation. Taken together, the data show that the newly established murine TTS model accurately recapitulates the human syndrome, and reveal that myocardial pro-inflammatory pathways are less activated in female mice than in male mice (Fig. 3c).

### Calcineurin-driven myocardial inflammation in eTTS

To identify potential drug targets in the more severely affected male mice, we analyzed gene regulation overlap with a freely available drug signature database (Tan Lab DSigDB version 1.0) and found the highest number of overlapping genes with valproic acid, copper sulfate and CsA (Extended Data Fig. 3e). Due to the QT-prolongation capacity of valproic acid and the observed inflammatory myocardial gene expression phenotype after EPI, we conducted a follow-up analysis involving pathways with opposite regulation from our gene expression network with CsA and found a considerable overlap of the gene set CICLOSPORIN_HL60_DOWN (Fig. 3d), indicative of a potential therapeutic effect of CsA on the myocardium in the setting of eTTS. Left ventricular immunoblotting revealed an upregulation of calcineurin A protein expression with particular upregulation of regulator of calcineurin 1 isoform 4 (RCAN1-4), a well-known marker of calcineurin activity in male but not female mice after EPI^19^ (Fig. 3e–g and Extended Data Fig. 3f). Calcineurin phosphorylation at serine 411 (p-Cn(Ser411)) is mainly driven by CaMKII, leading to the inhibition of calcineurin^17,25,26^. Intriguingly, the extent of p-Cn(Ser411) after injection with EPI was more pronounced in female mice than in male mice, counter to RCAN1-4 expression, pointing to a potential functional relationship between CaMKII activation and calcineurin inhibition in eTTS. Taken together, these findings are suggestive of pro-inflammatory myocardial calcineurin signaling in TTS.

### A new anti-inflammatory treatment strategy for TTS

**Fig. 4:**
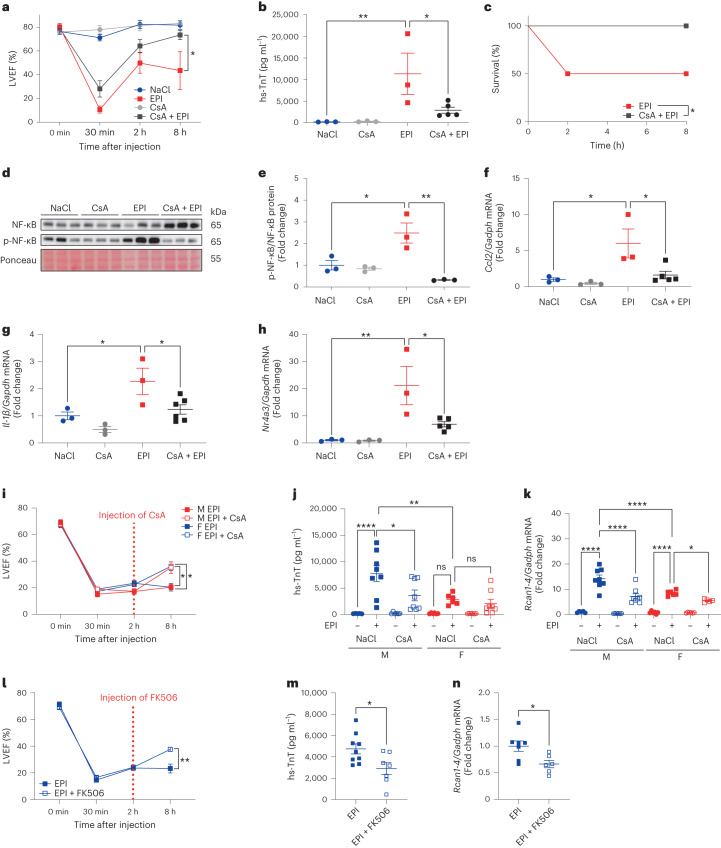
A new anti-inflammatory treatment strategy for TTS. **a**–**c**, Kinetics of LVEF 30 min after NaCl or EPI (NaCl, *n* = 3; CsA + NaCl, *n* = 3; EPI, *n* = 6; CsA + EPI, *n* = 6; *P = 0.049 (30 min–8 h)) (**a**), plasma hs-TnT levels at 24 h (NaCl, *n* = 3; CsA, *n* = 3; EPI, *n* = 3; CsA + EPI, *n* = 5; **P = 0.0024 and *P = 0.012) (**b**) and survival at 24 h (NaCl, *n* = 3; CsA, *n* = 3; EPI, *n* = 6; CsA + EPI, *n* = 6; *P = 0.024) (**c**) in 8-week-old male C57BL6/N mice, pretreated with a single dose of 10 mg kg^−1^ CsA 30 min before NaCl or EPI. **d**, Left ventricular immunoblotting of NF-κB p65 and its phosphorylation at Ser536 (p-NF-κB) at 8 h (*n* = 3 in each group). **e**, Relative quantification of left ventricular immunoblotting of NF-κB p65 and its phosphorylation at Ser536 (p-NF-κB) at 8 h (*n* = 3 in each group; *P = 0.014, **P = 0.001). **f**–**h**, mRNA per *Gadph* expression of left ventricular *Ccl2* (NaCl, *n* = 3; EPI, *n* = 3; CsA, *n* = 3; CsA + EPI, *n* = 5; both *P = 0.01) (**f**), left ventricular *Il-1β* (NaCl, *n* = 3; EPI, *n* = 3; CsA, *n* = 3; CsA + EPI, *n* = 6; NaCl versus EPI *P = 0.01, EPI versus CsA + EPI *P = 0.02) (**g**) and left ventricular *Nr4a3* (NaCl, *n* = 3; EPI, *n* = 3; CsA, *n* = 3; CsA + EPI, *n* = 5; *P = 0.011 and **P = 0.002) (**h**) at 8 h after single preventive CsA treatment. **i**–**k**, Kinetics of LVEF (M EPI, *n* = 7; M EPI + CsA, *n* = 7; F EPI + CsA, *n* = 7; F EPI, *n* = 6; M EPI versus M EPI + CsA *P = 0.013 and F EPI versus F EPI + CsA *P = 0.023) (**i**), hs-TnT (M NaCl, *n* = 5; M CsA, *n* = 6; M EPI, *n* = 8; M EPI + CsA, *n* = 8; F EPI + CsA, *n* = 8; F NaCl, *n* = 6; F CsA, *n* = 6; F EPI, *n* = 6; *P = 0.013, **P = 0.0048, ****P < 0.0001) (**j**) and *Rcan1-4* mRNA (M NaCl, *n* = 5; F EPI + CsA, *n* = 5; M CsA, *n* = 6; F NaCl, *n* = 6; F CsA, *n* = 6; F EPI, *n* = 6; M EPI, *n* = 8; M EPI + CsA, *n* = 8; *P = 0.024, ****P < 0.0001) (**k**) 8 h after NaCl and/or EPI with or without 30 mg kg^−1^ CsA at 2 h in 12-week-old male (2.5 mg kg^−1^ EPI) and female (3.5 mg kg^−1^ EPI) mice. **l**–**n**, Kinetics of LVEF (*n* = 7 in each group, **P = 0.0019) (**l**), hs-TnT (EPI, *n* = 9; EPI + FK506, *n* = 7; *P = 0.0227) (**m**) and *Rcan1-4* mRNA (EPI, *n* = 7; EPI + FK506, *n* = 6; *P = 0.0193) (**n**) at 8 h after EPI with or without 10 mg kg^−1^ FK506 at 2 h in 12-week-old male mice. Data expressed as mean ± s.e.m., from multiple comparisons adjusted ANOVA (**b**–**d**, **i**–**k**), two-sided paired *t*-test (**a**,**h**), Student’s *t*-test (**l**–**n**) or log-rank test (**c**). Source data

**Extended Data Fig. 4:**
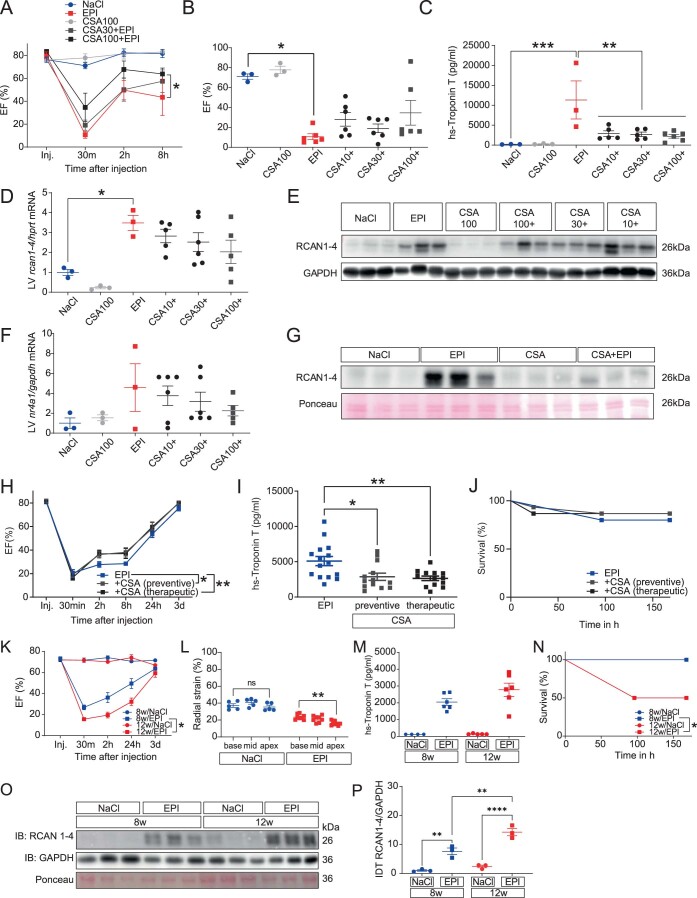
Calcineurin inhibition improves heart failure and myocardial damage. **(A)** Ejection fraction (EF) kinetics (n = NaCl, CsA100 3/group, EPI, CsA30 + EPI, CsA100 + EPI 6/group; *p = 0.036) and **(B)** EF at 30 min. (n = NaCl, CsA100 3/group, EPI, CsA100+, CsA30+, CsA10 + 6/group) after epinephrine (EPI) or NaCl0.9% (NaCl) in 8w old male mice pretreated with a single dose of 10- (CsA10+), 30- (CsA30+), or 100 mg (CsA100+)/kg body weight CsA 30 min before (*p = 0.0035). **(C)** Plasma high-sensitive Troponin T (hs-Troponin T) at 24 h (n = NaCl, EPI, CsA100 3/group, CsA100 + 6, CsA30+, CsA10 + 5/group; **p < 0.004, ***p = 0.0006). **(D)** Left ventricular tissue (LV) regulator of calcineurin 1-4 (RCAN1-4) mRNA expression (n = NaCl, EPI, CsA100 3/group, CsA30 + 6, CsA100+, CsA10 + 5/group; *p = 0.042) as well as **(E)** immunoblotting (IB) at 8 h. **(F)** LV nuclear receptor subfamily 4 group a member 1 (nr4a1) mRNA expression 8 h after EPI, NaCl, and CsA (n = NaCl, EPI, CsA100 3/group, CsA100 + 5, CsA30+, CsA10 + 6/group; p = 0.308). **(G)** LV RCAN1-4 IB of 8w old male mice after pretreatment with 10 mg/kg CsA 30 min. before NaCl or EPI from a separate experiment. **(H)** EF kinetics (n = 15/group; *p = 0.011, **p = 0.0079 at 2h-3d), **(I)** hs-Troponin T (n = EPI 15, CsA both 13/group; *p = 0.0128, **p = 0.0058), and **(J)** Kaplan Maier analysis (n = 15/group) of 12w old male mice with 10 mg/kg CsA 2 h before (preventive) or 30 min. after EPI (therapeutic) and subsequent CsA application twice per day (p = 0.221). **(K)** EF kinetics (n = 8w/NaCl 4, 8w/EPI, 12w/EPI 9/group, 12w/NaCl 6; *p = 0.027), **(L)** Radial strain from 12w old M C57BL6n mice 30 min after NaCl vs. EPI (n = NaCl 5, EPI 8; **p = 0.0013) **(M)** hs-Troponin T (n = 8w/NaCl 4, 8w/EPI, 12w/EPI 6/group, 12w/NaCl 5) and **(N)** Kaplan Maier analysis of M mice at 8- (8w) or 12 weeks of age (12w) with NaCl or EPI (n = NaCl 5, EPI 8; *p = 0.0167). **(O)** Corresponding western blotting of regulator of calcineurin 1-4 (RCAN1-4) and **(P)** quantification (IDT) per GAPDH (n = 3/group, **p = 0.0023, ****p < 0.0001). Data as mean ± SEM. Multiple comparisons adjusted ANOVA (B-D, F, I, L-M, P), two-sided paired T-Test (A, H, K), or Log-rank test (J, N). Source data

To further investigate the therapeutic efficacy of CsA, male mice were pretreated with a singular dose of 10, 30 or 100 mg kg^−1^ CsA 30 min before NaCl or EPI. CsA significantly improved cardiac function and ameliorated myocardial damage, with a beneficial effect on survival already at a dosage of 10 mg kg^−1^ but no additional benefits at higher dosages (Fig. 4a–c and Extended Data Fig. 4a–f). Immunoblotting revealed increased phosphorylation of nuclear factor kappa B (NF-κB) p65 at Ser536 8 h after EPI, which was blunted by additional CsA treatment (Fig. 4d,e). Also, left ventricular tissue qPCR confirmed the downregulation of mRNA expression of the representative CsA target gene chemokine ligand 2 (*Ccl2*) (Fig. 4f), of the pro-inflammatory cytokine interleukin-1β (*Il-1β*) (Fig. 4g), as well as of the overall top-ranked gene from GSEA (Extended Data Fig. 3a), the nuclear receptor subfamily 4 group A member 3 (*Nr4a3*) (Fig. 4h). However, at 8 h, only a mild dose-dependent decrease of RCAN1-4 mRNA and protein was observed (Extended Data Fig. 4d,e), indicative of weakening calcineurin suppression by CsA. Therefore, we continued CsA application twice daily in subsequent experiments. Continued CsA after initial pretreatment 2 h before EPI or therapeutic application 30 min after EPI in 8-week-old mice improved LVEF and significantly blunted hs-TnT compared to EPI, with significant RCAN1-4 reduction by CsA at 8 h (Extended Data Fig. 4g–i). However, after initial pretreatment 2 h before EPI or therapeutic application 30 min after EPI in 8-week-old mice, we observed a mild eTTS phenotype with low overall mortality (Extended Data Fig. 4j). Given that these mice were slightly younger (8 weeks) than those in our other experiments (10–12 weeks), we compared male 8-week-old and 12-week-old mice in a separate experiment and observed a marked impact of increased age on LVEF and mortality, with a trend towards increased myocardial damage in 12-week-old mice (Extended Data Fig. 4k–n). Apical impairment of radial strain was confirmed in 12-week-old mice (Extended Data Fig. 4l). Interestingly, immunoblotting revealed markedly increased left ventricular tissue RCAN1-4 in the more vulnerable 12-week-old mice, suggestive of an age-dependent exacerbation of calcineurin signaling that affects outcome in eTTS (Extended Data Fig. 4o,p). To also investigate the therapeutic potential of calcineurin inhibition in females and use an application timing with higher translational relevance, male and female mice were injected with EPI (males with 2.5 mg kg^−1^ and females with 3.5 mg kg^−1^) and treated with 30 mg kg^−1^ CsA 2 h later. With the increased dose of EPI in females, we found comparable AHF in male and female mice, with improvement of LVEF by CsA injected 2 h after EPI (Fig. 4i) and reduced myocardial damage (Extended Data Fig. 4j), in line with suppressed *Rcan1-4* expression (Extended Data Fig. 4k). To exclude mitochondrial permeability transition pore (MPTP) opening inhibition by CsA as the responsible mechanism of the observed therapeutic effects, mice were also injected with 10 mg kg^−1^ FK506 (tacrolimus) 2 h after EPI and displayed improved LVEF (Fig. 4l) and reduced plasma hs-TnT after 8 h (Fig. 4m), with blunted *Rcan1-4* expression (Fig. 4n). Taken together, we observed beneficial effects of preventive and therapeutic CsA application, with myocardial RCAN1-4 suppression and a blunted myocardial inflammatory response. Female mice required a higher dose of EPI for comparable AHF but even then exhibited lower myocardial damage and *Rcan1-4* expression compared to male mice. In line with this, the benefit of therapeutic CsA on LVEF was comparable in male and female mice, while amelioration of the exacerbated myocardial injury and *Rcan1-4* expression was more pronounced in male mice.

**Fig. 5:**
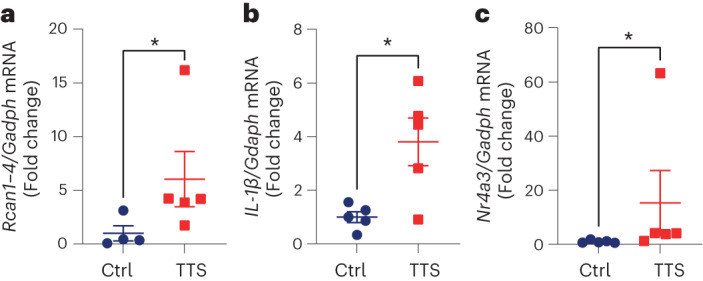
Calcineurin signaling in human TTS. **a**–**c**, In PBMCs from age- and sex-matched healthy controls (Ctrl) versus patients with TTS, the expression of *Rcan1-4* (Ctrl, *n* = 4; TTS, *n* = 5; *P = 0.031) (**a**), *Il-1β* (*n* = 5 in each group; *P = 0.015) (**b**) and *Nr4a3* (*n* = 5 in each group; **P = 0.009) (**c**) mRNA was significantly upregulated. Data expressed as mean ± s.e.m., from two-sided Mann–Whitney *U*-test (**a**,**c**) or Student’s *t*-test (**b**). Source data

To investigate whether this pathway is also regulated in biomaterial from patients with TTS, we analyzed peripheral blood mononuclear cells (PBMCs) from five patients with TTS and five healthy controls. Expression of the calcineurin regulator *Rcan1-4* (Fig. 5a), the pro-inflammatory gene *Il-1β* (Fig. 5b) and the top upregulated gene from our mouse model, *Nr4a3* (Fig. 5c), was significantly increased in PBMCs from patients with acute TTS. These data underscore the systemic nature of TTS, suggesting that non-cardiomyocytes may be useful for the diagnosis of TTS.

## Discussion

Due to the lack of a mouse model that closely recapitulates the clinical features of TTS, cause–effect relationship studies and genetic engineering approaches to understand the underlying mechanisms of TTS have so far been mostly unavailable. Here, we established an experimental mouse model that recapitulates the hallmarks of human TTS, including transient AHF, biomarker elevation, ECG changes and risk factors of impaired outcome. In this regard, the standardized setting of eTTS enabled us to detect early changes in gene expression and led to the identification of a considerable overlap of pro-inflammatory genes with CsA targets. Interestingly, the increase of left ventricular EPI levels was higher in male mice, while calcineurin phosphorylation, which has been suggested to inhibit calcineurin, was higher in female mice, suggesting the existence of a sex-specific cardioprotective mechanism in premenopausal mice. This cardioprotective mechanism is currently under investigation. We unmasked calcineurin-driven myocardial inflammation as a potential underlying mechanism and established early pharmacological calcineurin inhibition with CsA as a therapeutic approach to blunt features of TTS and improve survival. In line with these findings, PBMCs from patients with TTS indicate increased calcineurin activity, which may potentially reflect myocardial pathway activation.

### Epinephrine induces sex-specific reversible AHF in mice

Patients with TTS showed threefold higher plasma levels of circulating EPI compared to patients with acute myocardial infarction^6^, and case reports suggest that EPI is a potential trigger of TTS-like cardiac dysfunction^10^. Here, we observed that high-dose EPI (but not isoprenaline) is sufficient to induce TTS-like AHF in mice along with troponin elevation and acute ST-segment changes, thereby fulfilling the criteria of the human disease^27^. In line with our findings and the findings of others, EPI-induced and catecholamine-induced reversible AHF have been described in rats^11,12^. Consistent with our findings, isoprenaline has not been reported to cause immediate AHF in mice^13,28^, and since isoprenaline causes a secondary rise in plasma norepinephrine and may thereby stimulate α‐adrenoceptors indirectly, the possibility of selective β-adrenergic stimulation in vivo appears questionable^29^. Given that human TTS confers a sex-specific phenotype (i.e. it predominantly affects postmenopausal women, and men represent only 10% of patients with TTS but suffer from increased rates of prehospital cardiac arrest, increased troponin levels, higher occurrence of cardiogenic shock and increased mortality compared to women)^4,5,30,31^ we compared male and female C57BL/6N mice. In line with the clinical phenotype, we observed reduced cardiac function, increased myocardial damage and pro-inflammatory gene network upregulation, as well as markedly increased mortality in males. Moreover, we observed increased levels of corticosterone in eTTS, suggesting HPA-axis activation, and we interpreted left ventricular norepinephrine depletion as the result of adrenergic stimulation^32–35^. Our finding of left ventricular EPI upregulation despite precursor (dopamine, norepinephrine) depletion suggests enrichment due to EPI administration, with secondary cardiac sympathetic and HPA-axis activation. Given that left ventricular EPI upregulation was significantly enhanced in male mice and confirmed by dose titration, sex-specific cardiac catecholamine uptake or degradation is suggested. In patients with acute TTS, coronary sinus plasma norepinephrine concentrations were substantially increased compared to systemic plasma concentrations^36^, indicating an increased cardiac release. This finding is consistent with our observation of depleted left ventricular norepinephrine in eTTS and further supports our model. The importance of myocardial inflammation in TTS has been shown previously^15^ and is in accordance with our finding of increased myocardial inflammatory gene expression networks after eTTS. *Nr4a1*, a member of the nuclear receptor family *Nr4a*, has been implicated both in the pathogenesis of TTS in an in vitro induced pluripotent stem cell (iPSC) model^37^ and in vivo in transient cardiomyopathy or cardiac fatigue^38^. Due to their role in orchestrating adrenergic drive and inflammation and based on our GSEA ranking, further investigation of *Nr4a3* and of the stress-induced activating transcription factor 3 (*Atf3*) is warranted. In summary, sex-related differences in outcome may potentially be caused by increased myocardial EPI and exacerbated calcineurin-driven myocardial inflammation in male patients. A limitation of our study is the use of premenopausal female mice. Future investigations may consider including ovariectomized older mice to resemble the human condition more closely.

### Implication of calcineurin in eTTS

The sex-specific upregulation of RCAN1-4, a sensitive endogenous calcineurin reporter^25,39,40^, suggests that calcineurin has a role in discrepant myocardial inflammation and outcome in eTTS. Given that CaMKII has been shown to inhibit calcineurin in vivo^17,25,26^, the question arises as to whether CaMKII mediates beneficial effects in eTTS. This question will be addressed in future studies by combining eTTS with genetic mouse models.

**Fig. 6:**
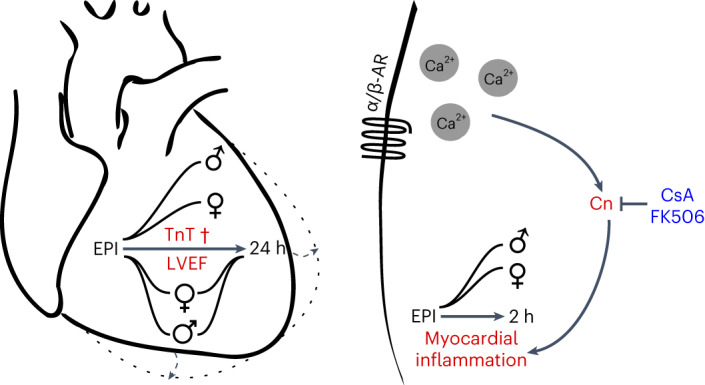
EPI injection recapitulates takotsubo syndrome in mice. We observed impaired outcomes in male compared to female mice, including mortality (†), reduction of LVEF and plasma TnT, with marked myocardial calcineurin activation and inflammation. Apical contractility was particularly blunted after EPI with elevated endsystolic left ventricular diameter, mimicking apical ballooning (dashed lines and arrows). Calcineurin inhibition by CsA or FK506 reduces the takotsubo phenotype in male and female mice. α/β-AR, α- or β-adrenergic receptor

We conducted preventive and therapeutic treatment of eTTS with CsA and observed significantly reduced myocardial damage and improved cardiac function. Given that CsA is also a potent inhibitor of MPTP opening, we also used FK506—which inhibits calcineurin but not the MPTP—in a therapeutic approach and were able to reproduce the protective effects, indicating that MPTP inhibition is likely not involved. We also observed a disadvantageous effect of higher murine age on LVEF, myocardial injury and survival (in 8-week-old versus 12-week-old mice). This is particularly interesting because we also observed an age-dependent increase in RCAN1-4, which may suggest that age may be a risk factor for adverse outcomes of TTS due to elevated calcineurin signaling^30^. However, the effect of actual aging remains to be investigated in this model. EPI caused increased myocardial RCAN1-4 and NF-κB p65 phosphorylation, which was reversed by CsA with a similar response of gene expression of inflammatory markers. Increased *Rcan1-4* expression in human PBMCs from patients with TTS compared to healthy controls underscores the systemic nature of the disease, suggesting that non-cardiomyocytes may also be useful for the diagnosis of TTS. Therefore, the data of this study point to an unexploited strategy for the treatment of TTS that involves CsA-mediated inhibition of the calcineurin pathway (Fig. 6). This concept is currently entering a DZHK (German Centre for Cardiovascular Research)-financed phase II clinical trial (Cyclosporin in TTS, CIT) to investigate the effect of CsA on myocardial damage in patients with TTS.

## Methods

### Experimental animals

The study conforms to the *Guide for the Care and Use of Laboratory Animals* published by the US National Institutes of Health (NIH, publication no. 85-23, revised 1985) and complies with all relevant ethical regulations. It was approved by the authorities of the Regierungspräsidium Karlsruhe, Germany (G-1/16, G-25/17, G-143/17, G-149/18 and G-95/18). Every effort was made to minimize the number of animals used and their suffering. Animals were housed with access to food and water ad libitum on a 12 h–12 h light–dark cycle at 21 °C and 50–60% humidity. For all experiments, male or female C57BL/6N mice were used. For some experiments, commercially available mice (C57BL/6N) were obtained from Janvier Labs. In experiments involving calcineurin inhibition, mice were injected intraperitoneally with 10, 30 or 100 mg kg^−1^ CsA, 10 mg kg^−1^ FK506 (tacrolimus) or 0.9% NaCl in a total volume of 200 µl before, 30 min or 2 h after the injection of EPI hydrochloride (EPI, Sanofi Aventis), as described below. If not indicated otherwise, mice were the same age (week) in the corresponding groups in each experiment (8–12 weeks).

### Echocardiography

Cardiac function was evaluated using 2D echocardiography at baseline and as indicated in the corresponding experiments, after NaCl or EPI injection under isoflurane volatile mask narcosis (1–3% vol) at a constant temperature of 38 °C (Fig. 1) or in awake mice (Figs. 2, 4 and 5), using a Visual Sonics Vevo 2100 with an MX550D transducer by an experienced investigator blinded to the animal’s group. Mice were shaved and left ventricular parasternal short-axis views were obtained in M-mode imaging at the papillary muscle level, as well as parasternal long-axis views. A cut-off of >400 bpm was used to avoid the confounding effects of narcosis and bradycardia on cardiac function^20^. Awake mice were trained to avoid stress in the first instance. Four consecutive beats were used to measure the left ventricular end-diastolic internal diameter (LVEDD), left ventricular end-systolic internal diameter (LVESD) and LVEF. Moreover, left ventricular parasternal long-axis (PSLAX) views were obtained for accurate quantification of LVEF. Additional left ventricular strain analysis was conducted in PSLAX with images acquired at >200 frames per second using Visual Sonics Vevo Strain Analysis.

### ECG telemetry recordings

In a random subset of male C57BL/6N mice, we performed telemetry recording of heart rate and blood pressure. After analgesia with 0.1 mg kg^−1^ (subcutaneous) of buprenorphine 1 h before the intervention, isoflurane narcosis (3% vol) was induced with subsequent subcutaneous implantation of transmitters according to the manufacturer’s recommendations (ETA-F10/X11, Data Sciences International). Analgesia was continued immediately after the intervention with carprofen (5 mg kg^−1^ (subcutaneous)) twice daily for 48 h with subsequent close monitoring for 7 d and a total recovery time of 2 weeks before starting measurements. During the subsequent experiments, ECG and blood pressure were recorded in mice undergoing volatile mask narcosis and in freely moving mice using a PhysioTel telemetry setup (DSI). Data were recorded and analyzed using Ponemah (DSI) software including Data Insights. Analysis was performed using the mean of 30 s intervals between the indicated time points (baseline, 15 m, 30 m, 2 h, 4 h, 6 h, 12 h, 24 h for blood pressure and baseline, 5 m, 10 m, 15 m, 20 m, 25 m, 30 m for R-wave amplitude and ST-segment elevation) according to conventional guidelines^41^.

### Experimental design of epinephrine-induced heart failure (eTTS)

To establish an easily reproducible mouse model of TTS, 10-week-old male C57BL/6N mice underwent isoflurane narcosis and baseline echocardiography with subsequent intraperitoneal injection of 0.9% NaCl, ascending doses of EPI (2, 2.5 and 5 mg kg^−1^ body weight) or isoprenaline (250 mg kg^−1^ body weight) diluted to a total volume of 100 µL and echocardiography after 30 min. The mice were killed 7 d later. The findings from this experiment resulted in our final protocol for eTTS. After baseline echocardiography, the mice were subjected to a single injection of 2.5 mg kg^−1^ body weight of EPI or NaCl under volatile mask narcosis (isoflurane 1–3% vol) at a constant body temperature of 38 °C to prevent hypertensive crisis. After 15 min, narcosis was terminated, and follow-up echocardiography was performed at 30 min, 2 h, 8 h, 24 h, 3 d and 7 d. At 24 h, facial vein blood was collected. The mice were killed by decapitation at different time points depending on their assigned group, and trunk blood was collected. The heart was isolated, the left and right ventricle were dissected and immediately snap-frozen in liquid nitrogen. Tissue was pulverized in a mortar and stored at −80 °C until further evaluation.

### Measurement of troponin T and corticosterone

Facial vein blood or trunk blood was collected from mice using hematocrit capillaries 24 h after the induction of eTTS. Whole blood was centrifuged at 14,000×*g* for 20 min at 4 °C. Supernatants were stored until further analysis at −80 °C. For quantification of infarct size, high-sensitivity cardiac troponin T (hs-cTnT) was measured using an automated Cobas Troponin T hs STAT Elecsys (Roche) as described before^22^. Corticosterone was measured using radioimmunoassay (RIA) at the Steroid Laboratory of University Hospital Heidelberg (Department of Pharmacology) as described previously^42^. In brief, 10 μl of plasma were added to 100 μl of 5% ethanol and tritium-labeled corticosterone, and mixture extraction was performed with 1 ml of cyclohexane/dichloromethane (2:1). The extract was separated, dried, dissolved in 1 ml of 5% ethanol and quantified by RIA. The antisera used were raised in the Steroid laboratory of University Hospital Heidelberg (Department of Pharmacology) and extensively characterized, especially for cross-reactivity with potentially interfering endogenous and exogenous steroids. Each result was corrected for individually determined procedural loss.

### Measurement of plasma and left ventricular catecholamines

Cardiac tissue was weighted and subsequently homogenized in an ice-cold solution (0.01 M HCl, 1 mM EDTA, 4 mM sodium disulfide). Whole blood was centrifuged at 14,000×*g* for 20 min at 4 °C and diluted 1:40 with the same ice-cold solution. Measurements were performed using high-performance liquid chromatography (HPLC) coupled with electrochemical detection (potential 0.48–0.6 V, range 0–20 nA) at the Central Laboratory of University Hospital Heidelberg (Department of Endocrinology and Clinical Chemistry), as previously described^35^. Calibration was performed with the use of an internal standard (dihydroxybenzylamine, Chromsystems). After washing the samples three times with washing buffer (3 × 1 ml, Chromsystems) as well as centrifugation, 120 µl of elution buffer were added for 5 min, followed by additional centrifugation with the addition of 20 µl 1 M HCl before quantification. For each quantification, 50 µl were automatically injected. The flow rate was 1 ml min^−1^. The detection limit for dopamine was 60 ng l^−1^ (391.8 pmol l^−1^), for norepinephrine 50 ng l^−1^ (295.5 pmol l^−1^) and for epinephrine 50 ng l^−1^ (273 pmol l^−1^). Results were calculated in pmol l^−1^ for plasma catecholamines and pg mg^−1^ for tissue levels.

### RNA extraction and qPCR

Total RNA was isolated from homogenized left ventricular tissue using TRIzol (Invitrogen). Total RNA was digested with DNase, and cDNA synthesis of 500 ng of RNA was carried out using a SuperScript first-strand synthesis system for RT–PCR (Invitrogen). qPCR was performed with Universal ProbeLibrary (Roche) using TaqMan Universal PCR Mastermix (Applied Biosystems) and detection on a 7500 Fast Cycler (Applied Biosystems).

### RNA sequencing

Strand-specific TruSeq mRNA libraries were prepared at the Cologne Center for Genomics, Cologne, Germany (Ribo-Zero, 2 × 75 nt, >30 M fragments). Libraries were paired-end sequenced on an Illumina HiSeq 3000 instrument. We used Flexbar to remove adaptor sequences and low-quality regions from FASTQ files^43^. Reads > 18 bp were retained and mapped against the murine 45 S ribosomal RNA precursor sequence (BK000964.3) to remove rRNA contaminant reads. We used the mouse genome sequence and annotation (GRCm38_90) together with the splice-aware STAR read aligner (release 2.5.1b) to map our short reads^44^. Afterwards, transcriptome analyses were carried out with the cufflinks package^45^. GSEA was conducted using GSEA 4.0.3 software and Molecular Signatures Database (MSigDB 7.2) from the Broad Institute^46,47^. Gene overlap network design was conducted using the EnrichmentMap plugin^48^ for Cytoscape software (version 3.8.0) (refs. ^49,50^) and the collection of annotated drug gene sets from the Drug SIGnatures DataBase (DSigDB 1.0) from Tanlab^51^.

### Immunoblotting

Extracts from left ventricular tissue were isolated, and western blot analysis was performed according to protocols described previously^52^. Primary antibodies used were directed against total CaMKII (1:1,000, no. 611293, lot 9343525, BD Biosciences), calcineurin A (Cn) (1:1,000, no. 07-1491, lot 3792860, Millipore), phospho-calcineurin A (p-Cn) at Ser411 (p-Cn(Ser411)) (1:1,000, Pineda Antibody Service), RCAN1-4 (1:1,000, a kind gift from Timothy McKinsey), NF-κB (1:1,000, no. D14E12, lot 16, Cell Signaling) and phospho-NF-κB p65 (Ser536) (1:1,000, no. 3033 S, lot 17, Cell Signaling). Antibodies were diluted with 5% skimmed milk (no. T145.2, Carl Roth). Primary antibody incubation was followed by incubation with the corresponding HRP-conjugated secondary anti-mouse (1:5,000, no. 1031-05, lot H0021-MA82, Southern Biotech) and anti-rabbit (1:5,000, no. 4050-05, lot A1420-SQ21E, Southern Biotech) antibodies and detection with enhanced chemiluminescence (Santa Cruz, sc-2048). Western blots were developed using Fusion FX7 Edge software (Vilber Lourmat). Western blot densitometry was assessed using GelQuant 1.8.2 (BiochemLabSolutions).

### Human samples

Venous blood (70 ml) was collected from five patients diagnosed with acute TTS (*n* = 5, mean age 69.4 ± 3.8 years (s.e.m.)) as well as five healthy control subjects (*n* = 5, mean age 53.3 ± 2.86 years (s.e.m.)) at Aberdeen Royal Infirmary, UK. Patients with TTS were included based on the InterTAK Diagnostic Criteria^1^, and the diagnosis was confirmed using gadolinium-enhanced cardiovascular magnetic resonance. PBMCs were isolated from fresh peripheral venous blood from patients upon presentation to the emergency room using standard Ficoll-Paque (Ficoll-Paque Plus, GE Healthcare) centrifugation separation with subsequent storage at −80 °C until mRNA and protein analysis. Patients were recruited at the Cardiovascular and Diabetes Centre, School of Medicine and Dentistry, University of Aberdeen, UK. The study was approved by the South Central – Hampshire B Research Ethics Committee, and all patient samples were collected after informed consent without participant compensation (EC ref. no. 20/SC/0305).

### Statistical analysis

Results are expressed as mean ± s.e.m. Normal distribution was tested using the Kolmogorov–Smirnov test. Statistical analysis included one-way analysis of variance (ANOVA) or Kruskal–Wallis test followed by Bonferroni, Sidak, or Dunn’s post-hoc test, respectively. Survival analysis was conducted using a log-rank test. An unpaired or paired Student’s *t*-test or Mann–Whitney *U*-test were used when appropriate. Statistical analysis was performed using GraphPad Prism 9 (GraphPad Software). A *P* value of <0.05 was considered statistically significant.

### Reporting summary

Further information on research design is available in the Nature Portfolio Reporting Summary linked to this article.

### Supplementary information

Reporting Summary

### Source data

Source Data Fig. 1 Statistical source data for Fig. 1.

Source Data Fig. 2 Statistical source data for Fig. 2.

Source Data Fig. 3 Statistical source data for Fig. 3 and uncropped western blot of Fig. 3e.

Source Data Fig. 4 Statistical source data for Fig. 4 and uncropped western blot of Fig. 4d.

Source Data Fig. 5 Statistical source data for Fig. 5.

Source Data Extended Data Fig. 1 Statistical source data for Extended Data Fig. 1.

Source Data Extended Data Fig. 2 Statistical source data for Extended Data Fig. 2.

Source Data Extended Data Fig. 3 Statistical source data for Extended Data Fig. 3.

Source Data Extended Data Fig. 4 Statistical source data for Extended Data Fig. 4 and uncropped western blots of Extended Data Figs. 4e, 4g and Extended Data Fig. 4o.

## Data Availability

The authors declare that the data supporting the findings of this study are available within the paper and its supplementary information. RNA sequencing data are available from ENA and BioStudies under accession number E-MTAB-13031. The following publicly available data (or datasets) were used: murine 45 S ribosomal RNA precursor sequence (BK000964.3), mouse genome sequence and annotation (GRCm38_90) together with the splice-aware STAR read aligner (release 2.5.1b) (ref. ^44^), and the cufflinks package version 2.2.1. GSEA was conducted with the GSEA 4.0.3 software and the Molecular Signatures Database (MSigDB 7.2, Broad Institute)^46,47^. Gene overlap network design was conducted via the EnrichmentMap plugin for the Cytoscape software (3.8.0) (refs. ^49,50^) and the collection of annotated drug gene sets from the Drug SIGnatures DataBase (DSigDB 1.0, Tanlab)^51^.
