# Supplementary material for: Calcineurin signaling promotes takotsubo syndrome
Source: Nat Cardiovasc Res. 2023 Jul 13;2(7):645–55. doi: 10.1038/s44161-023-00296-w (PMC11358029; doi:10.1038/s44161-023-00296-w)
Supplement: Supplementary file 1 — Reporting Summary [file 44161_2023_296_MOESM1_ESM.pdf]

nature portfolio

Corresponding author(s): Johannes Backs
Last updated by author(s): May 22, 2023

Reporting Summary

Nature Portfolio wishes to improve the reproducibility of the work that we publish. This form provides structure for consistency and transparency in reporting. For further information on Nature Portfolio policies, see our Editorial Policies and the Editorial Policy Checklist.

Statistics

For all statistical analyses, confirm that the following items are present in the figure legend, table legend, main text, or Methods section.

n/a	Confirmed	
☐	☒	The exact sample size (n) for each experimental group/condition, given as a discrete number and unit of measurement
☐	☒	A statement on whether measurements were taken from distinct samples or whether the same sample was measured repeatedly
☐	☒	The statistical test(s) used AND whether they are one- or two-sided<br>*Only common tests should be described solely by name; describe more complex techniques in the Methods section.*
☒	☐	A description of all covariates tested
☐	☒	A description of any assumptions or corrections, such as tests of normality and adjustment for multiple comparisons
☐	☒	A full description of the statistical parameters including central tendency (e.g. means) or other basic estimates (e.g. regression coefficient) AND variation (e.g. standard deviation) or associated estimates of uncertainty (e.g. confidence intervals)
☐	☒	For null hypothesis testing, the test statistic (e.g. F, t, r) with confidence intervals, effect sizes, degrees of freedom and P value noted<br>*Give P values as exact values whenever suitable.*
☒	☐	For Bayesian analysis, information on the choice of priors and Markov chain Monte Carlo settings
☒	☐	For hierarchical and complex designs, identification of the appropriate level for tests and full reporting of outcomes
☒	☐	Estimates of effect sizes (e.g. Cohen's d, Pearson's r), indicating how they were calculated

Our web collection on statistics for biologists contains articles on many of the points above.

Software and code

Policy information about availability of computer code

Data collection	Western blots were developed using Fusion FX7 Edge software (Vilber Lourmat GmbH, 88436 Eberhardzell, Germany). Echocardiography and strain analysis was conducted using VevoLAB 5.5.1 software (FUJIFILM VisualSonics, Inc., 3080 Toronto, Canada). Quantitative PCR was measured on a 7500 Fast Cycler with LightCycler® 480 Software 1.5.1.62 SP3 (Roche Diagnostics, Corp. 46250 Indianapolis, USA). Telemetry data was recorded and analyzed by Ponemah (DSI) software including Data Insights™.
Data analysis	For RNA-Seq. data extraction and analysis we used RStudio 1.4 and the STAR read aligner (release 2.5.1b) to map our short reads. Gene set enrichment analysis was conducted utilizing the GSEA 4.0.3 software and Molecular Signatures Database (MSigDB 7.2) (Broad Institute, USA). Western blot densitometry was assessed using GelQuant 1.8.2 (BiochemLabSolutions). Gene overlap network design was conducted via the EnrichmentMap plugin for the Cytoscape software (3.8.0) and the collection of annotated drug gene sets from the Drug SIGnatures DataBase (DSigDB 1.0, Tanlab, USA). Statistical analysis was conducted with Prism 9 GraphPad Software (92108 San Diego, USA). Figures were designed using Inkscape 1.0.2-2 (Open Source Software licensed under the GPL) and Adobe Illustrator® (Creative Cloud®, Adobe Inc., USA).

For manuscripts utilizing custom algorithms or software that are central to the research but not yet described in published literature, software must be made available to editors and reviewers. We strongly encourage code deposition in a community repository (e.g. GitHub). See the Nature Portfolio guidelines for submitting code & software for further information.

Data

Policy information about availability of data

All manuscripts must include a data availability statement. This statement should provide the following information, where applicable:

- Accession codes, unique identifiers, or web links for publicly available datasets
- A description of any restrictions on data availability
- For clinical datasets or third party data, please ensure that the statement adheres to our policy

The authors declare that the data supporting the findings of this study are available within the paper and its Supplementary Information. RNA-sequencing data are available from ENA/BioStudies (accession number E-MTAB-13031). The following publicly available data(sets) were used: murine 45S ribosomal RNA precursor sequence (BK000964.3), mouse genome sequence and annotation (GRCm38_90) together with the splice-aware STAR read aligner (release 2.5.1b), the cufflinks package version 2.2.1 (cuffdiff -p 2 --min-reps-for-js-test 2 --dispersion-method per-condition --output-dir cuffdiff_ref --library-type fr-firststrand --use-sample-sheet /biodb/genomes/mus_musculus/GRCm38_90/GRCm38.90.gtf sample_sheet_full.txt). Gene set enrichment analysis was conducted with the GSEA 4.0.3 software and the Molecular Signatures Database (MSigDB 7.2, Broad Institute, USA). Gene overlap network design was conducted via the EnrichmentMap plugin for the Cytoscape software (3.8.0) and the collection of annotated drug gene sets from the Drug SIGnatures DataBase (DSigDB 1.0, Tanlab, USA).

Human research participants

Policy information about studies involving human research participants and Sex and Gender in Research.

Reporting on sex and gender	Sex was considered in study design. Sex-specific analysis was conducted in commercially available C57BL6N mice and the results are reported in the manuscript.
Population characteristics	All TTS patients met the inclusion criteria (InterTAK Diagnostic Criteria and confirmation of a diagnosis of TTS by Gadolinium enhanced CMR) (mean age 69.4±3.8 SEM, n=5). Controls were healthy subjects (mean age 53.3±2.86 SEM, n=5). For TTS patients, mean time to sampling was 4 days and baseline EF from MRI was 48.4±2.16 SEM. TTS patients were all female and suffered from the following comorbidities: hypertension (n=2), Diabetes mellitus (n=1), psychiatric disease (n=1), atrial fibrillation (n=1). TTS patients were under treatment with the following medications: Betablockers (n=3), ACE-inhibitors (n=2), Angiotensin receptor blockers (n=1), anticoagulation (n=1), platelet inhibitors (n=3), statins (n=3), antidepressants (n=1). No TTS patient was treated with calcium inhibitors. Healthy female (n=4) and male (n=1) controls did not suffer from any known comorbidities and did not take any medication.
Recruitment	Patients were recruited upon presentation to the emergency room at the Cardiovascular and Diabetes Centre, School of Medicine and Dentistry, University of Aberdeen, United Kingdom after fulfilling the inclusion criteria. After confirmation of the diagnosis of TTS by CMR sampling of pbmcs was conducted with a mean delay of 4 days after symptom onset. The relatively late timepoint of sampling after disease onset may have weakened inflammatory gene expression in TTS patient pbmcs.
Ethics oversight	Patients were recruited at the Cardiovascular and Diabetes Centre, School of Medicine and Dentistry, University of Aberdeen, United Kingdom. The study was approved by the South Central – Hampshire B Research Ethics Committee and all patient samples were collected upon informed consent without participant compensation (EC ref. no. 20/SC/0305).

Note that full information on the approval of the study protocol must also be provided in the manuscript.

Field-specific reporting

Please select the one below that is the best fit for your research. If you are not sure, read the appropriate sections before making your selection.

☒ Life sciences ☐ Behavioural & social sciences ☐ Ecological, evolutionary & environmental sciences

For a reference copy of the document with all sections, see nature.com/documents/nr-reporting-summary-flat.pdf

Life sciences study design

All studies must disclose on these points even when the disclosure is negative.

Sample size	Sample size calculatios were based on murine EF and mortality from previous pilot experiments. If sample size could only be guessed, a pilot design was chosen yielding n=6 replicates per group.
Data exclusions	Data exclusions were not performed.
Replication	Animal experiments were not per se replicated (due to animal welfare reasons). However, similar independent experiments were performed reproducing at least once the obtained results.
Randomization	Animals were randomly assigned to experimental groups.

Blinding	Investigators were blinded regarding group allocation.

Reporting for specific materials, systems and methods

We require information from authors about some types of materials, experimental systems and methods used in many studies. Here, indicate whether each material, system or method listed is relevant to your study. If you are not sure if a list item applies to your research, read the appropriate section before selecting a response.

Materials & experimental systems

n/a	Involved in the study
☐	☒ Antibodies
☒	☐ Eukaryotic cell lines
☒	☐ Palaeontology and archaeology
☐	☒ Animals and other organisms
☒	☐ Clinical data
☒	☐ Dual use research of concern

Methods

n/a	Involved in the study
☒	☐ ChIP-seq
☒	☐ Flow cytometry
☒	☐ MRI-based neuroimaging

Antibodies

Antibodies used	Primary ABs: anti-CaMKII (1:1000, No. 611293, Lot 9343525 BD Biosciences), anti-calcineurin Pan A (1:1000, No. 07-1491, Lot 3792860, Millipore), anti-Ser411-phospho-calcineurin (1:1000, generated by Pineda antibodies, 69120 Heidelberg, Germany), anti-rcan1.4 (1:1000, Dr. Timothy McKinsey, Denver, USA), anti-nf-κb (1:1000, No. D14E12, Lot 16, Cell Signaling), anti-ser536-phospho-nf-κb p65 (1:1000, No. 3033S, Lot 17, Cell Signaling).<br>Secondary ABs: HRP-conjugated anti-mouse (1:5000, No. 1031-05, Lot H0021-MA82, Southern Biotech) and anti-rabbit (1:5000, No. 4050-05, Lot A1420-SQ21E, Southern Biotech).
Validation	The utilized anti-CaMKII (No. 611293, Lot 9343525 BD Biosciences), anti-Ser411-phospho-calcineurin (generated by Pineda antibodies, 69120 Heidelberg, Germany), and anti-rcan1.4 (Dr. Timothy McKinsey, Denver, USA; Proc Natl Acad Sci U S A. 2004 Mar 2;101(9):2870-5) primary antibodies have been used by us and others and published before (Circulation. 2014 Oct 7;130(15):1262-73). The anti-Ser411-phospho-calcineurin antibody has been used before to show the selective CaMKII-driven calcineurin phosphorylation at Ser411. We observed defective Ser411 phosphorylation in CaMKII KO mice with the utilized antibody at 60kDa. Validation for WB use of the anti-calcineurin Pan A (No. 07-1491, Lot 3792860, Millipore) has been conducted by Millipore in Mouse brain lysate. The anti-nf-κb (No. D14E12, Lot 16, Cell Signaling) and anti-ser536-phospho-nf-κb p65 (No. 3033S, Lot 17, Cell Signaling) antibodies have also both been published (Ann Transl Med. 2021 Jun;9(11):920; Int J Mol Med. 2021 Apr;47(4):39).

Animals and other research organisms

Policy information about studies involving animals; ARRIVE guidelines recommended for reporting animal research, and Sex and Gender in Research

Laboratory animals	Male and female C57BL6N mice age 8-12 weeks
Wild animals	No wild animals were used in this study.
Reporting on sex	Sex was considered in study design and the results are reported in the study.
Field-collected samples	No field-collected samples were used in this study.
Ethics oversight	The study conforms to the Guide for the Care and Use of Laboratory Animals published by the US National Institutes of Health (NIH Publication No. 85-23, revised 1985) and was approved by the authorities of the Regierungspräsidium Karlsruhe, Germany (G-1/16, G-25/17, G-143/17, G-149/18, and G-95/18).

Note that full information on the approval of the study protocol must also be provided in the manuscript.
