# Supplementary figures and images for: Calcineurin signaling promotes takotsubo syndrome
Source: Nat Cardiovasc Res. 2023 Jul 13;2(7):645–55. doi: 10.1038/s44161-023-00296-w (PMC11358029; doi:10.1038/s44161-023-00296-w)

Figure 3E

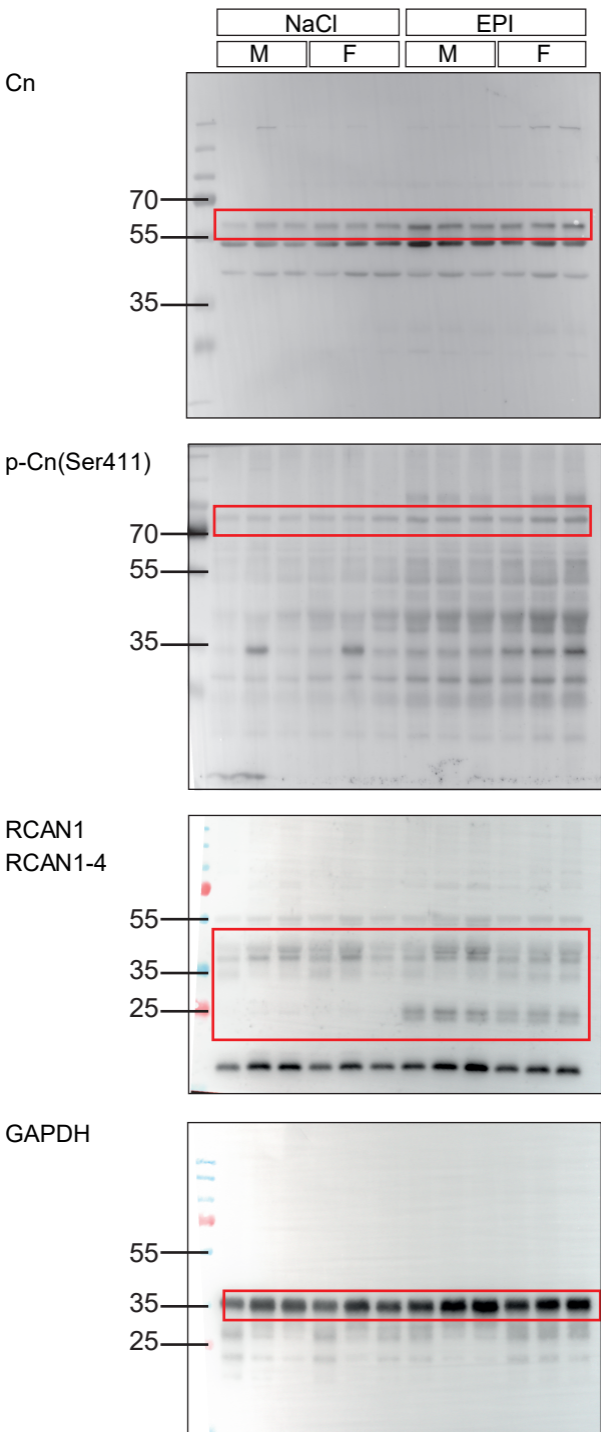

Supplement: Supplementary file 4 — Statistical source data for Fig. 3 and uncropped western blot of Fig. 3e. [file 44161_2023_296_MOESM4_ESM.zip › Source_Data_Blots_Figure3E.pdf]

Figure 4D

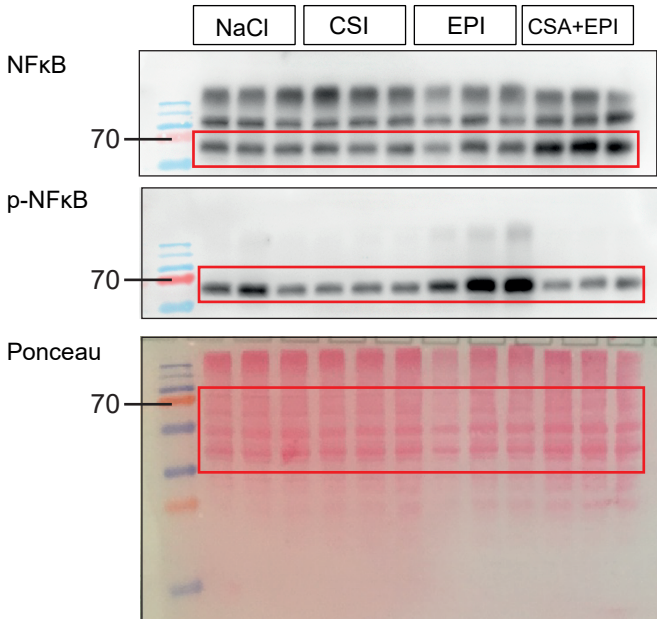

Supplement: Supplementary file 5 — Statistical source data for Fig. 4 and uncropped western blot of Fig. 4d. [file 44161_2023_296_MOESM5_ESM.zip › Source_Data_Blots_Figure4D.pdf]

Extended Data Figure 4E

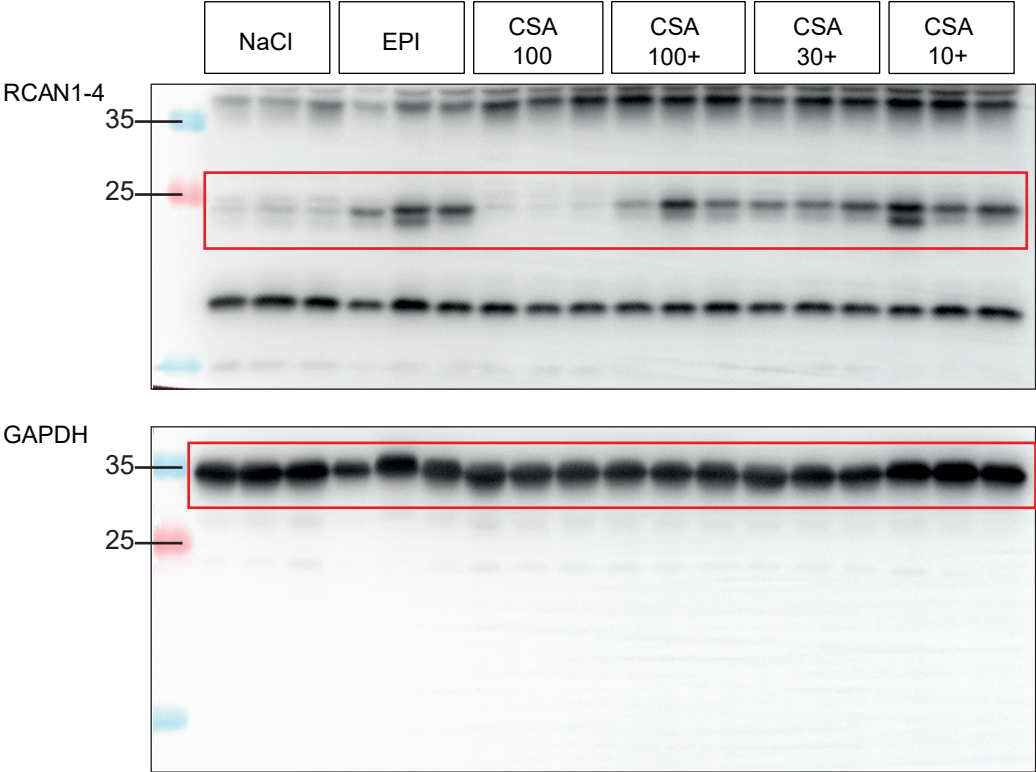

Supplement: Supplementary file 10 — Statistical source data for Extended Data Fig. 4 and uncropped western blots of Extended Data Figs. 4e, 4g and Extended Data Fig. 4o. [file 44161_2023_296_MOESM10_ESM.zip › Source_Data_Blots_ED_Figure4E.pdf]

Extended Data Figure 4G

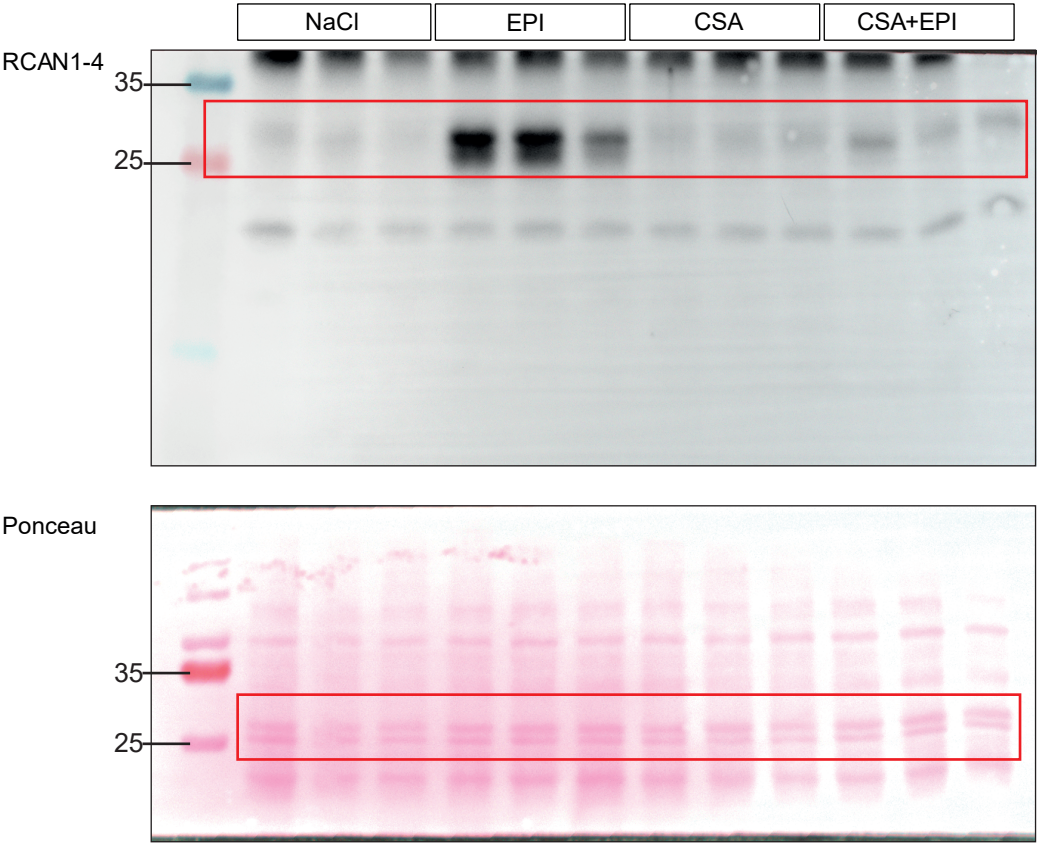

Supplement: Supplementary file 10 — Statistical source data for Extended Data Fig. 4 and uncropped western blots of Extended Data Figs. 4e, 4g and Extended Data Fig. 4o. [file 44161_2023_296_MOESM10_ESM.zip › Source_Data_Blots_ED_Figure4G.pdf]

# Extended Data Figure 4O

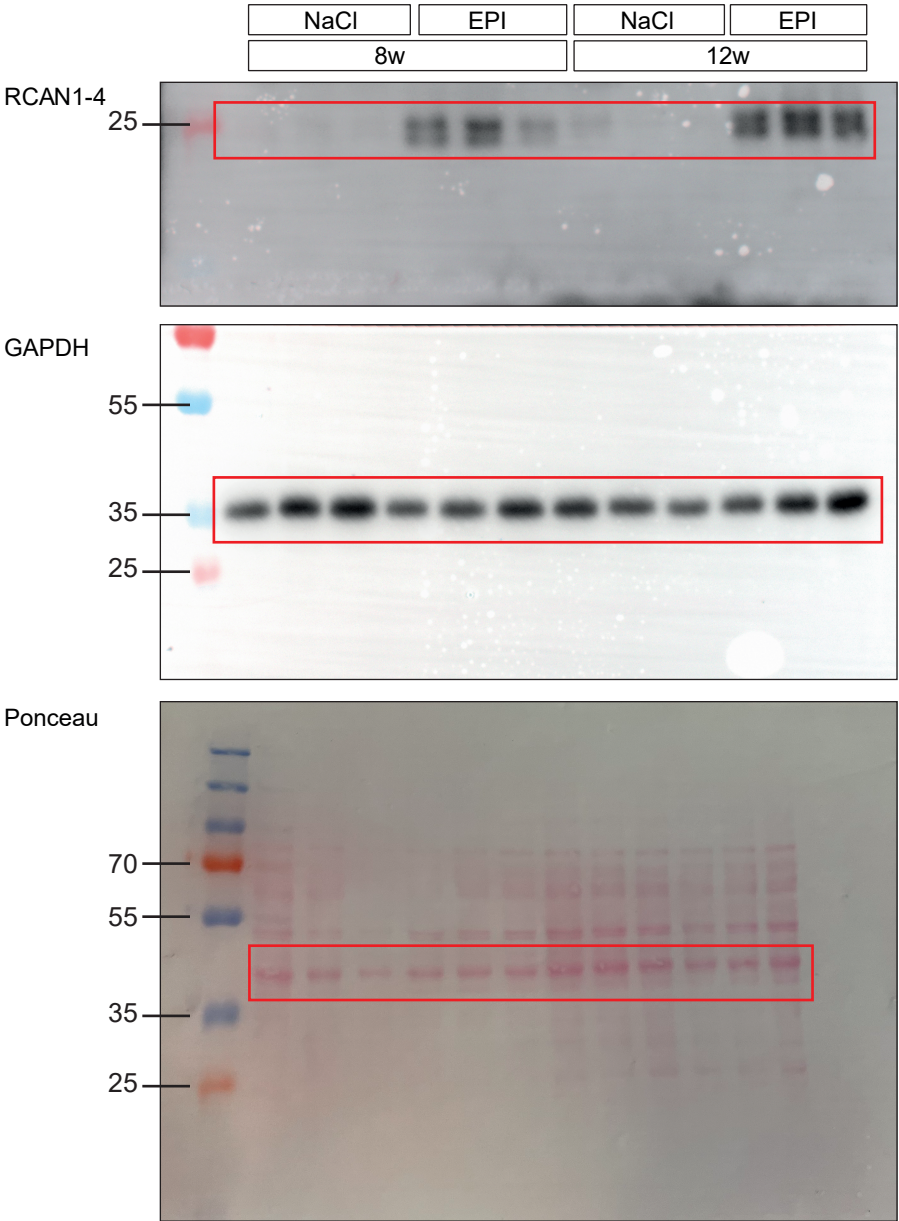

Supplement: Supplementary file 10 — Statistical source data for Extended Data Fig. 4 and uncropped western blots of Extended Data Figs. 4e, 4g and Extended Data Fig. 4o. [file 44161_2023_296_MOESM10_ESM.zip › Source_Data_Blots_ED_Figure4O.pdf]
